# Lung function in HIV-infected children and adolescents

**DOI:** 10.1186/s41479-018-0050-9

**Published:** 2018-06-25

**Authors:** Leah N. Githinji, Diane M. Gray, Heather J. Zar

**Affiliations:** Department of Paediatrics and Child Health, Red Cross War Memorial Children’s Hospital and MRC Research Unit on Child and Adolescent Health, University of Cape Town, Rondebosch, Klipfontein Road 7700, Cape Town, South Africa

**Keywords:** HIV, Lung function, Children, Adolescents

## Abstract

**Background:**

The advent of antiretroviral therapy has led to the improved survival of human immunodeficiency virus (HIV)-infected children to adulthood and to HIV becoming a chronic disease in older children and adolescents. Chronic lung disease is common among HIV-infected adolescents. Lung function measurement may help to delineate the spectrum, pathophysiology and guide therapy for HIV-related chronic lung disease.

**Aim:**

The aim of this study was to review the available data on the spectrum and determinants of lung function abnormalities and the impact of antiretroviral therapy on lung function in perinatally HIV-infected children and adolescents.

**Methods:**

Electronic databases “PUBMED”, “African wide” and “CINAHL” via EBSCO Host, using the MeSH terms “Respiratory function” AND “HIV” OR “Acquired Immunodeficiency Syndrome” AND “Children” OR “Adolescents”, were searched for relevant articles on lung function in HIV-infected children and adolescents. The search was limited to English language articles published between January 1984 and September 2017.

**Results:**

Eighteen articles were identified, which included studies from Africa, the United States of America (USA) and Italy, representing 2051 HIV-infected children and adolescents, 68% on antiretroviral therapy, aged from 50 days to 24 years. Lung function abnormalities showed HIV-infected participants had increased irreversible lower airway expiratory obstruction and reduced functional aerobic impairment on exercise, compared to HIV-uninfected participants. Mosaic attenuation, extent of bronchiectasis, history of previous pulmonary tuberculosis or previous lower respiratory tract infection and cough for more than 1 month were associated with low lung function. Pulmonary function tests in children established on antiretroviral therapy did not show aerobic impairment and had less severe airway obstruction.

**Conclusion:**

There is increasing evidence that HIV-infected children and adolescents have high prevalence of lung function impairment, predominantly irreversible lower airway obstruction and reduced aerobic function.

## Background

Improved survival of perinatally human immunodeficiency virus (HIV)-infected children to adolescence has occurred with the scale-up of pediatric antiretroviral therapy (ART) and prevention of mother-to-child transmission (PMTCT) programs. This has led to a large cohort of youth living with vertically transmitted HIV in sub-Saharan Africa [1]. Of the 2.3 million children living with HIV globally, 43% are on ART [2, 3]. In 2016, 7 million people were reported to be living with HIV in South Africa, of which 350,000 were between 10 and 19 years old [2].

HIV-related chronic lung disease (CLD) is a major cause of morbidity and mortality [4, 5]. In the post-ART era, the spectrum of CLD has changed from lymphocytic interstitial pneumonitis (LIP) being most predominant to bronchiolitis obliterans and bronchiectasis being more prevalent patterns [5, 6]. The spectrum of chronic lung disease in HIV infection has broad clinical phenotypes. For example, bronchiolitis obliterans may present as an obstructive pattern on spirometry [5], while chronic *Pneumocystis jirovecii* pneumonia (PCP), pulmonary tuberculosis (TB), bronchiectasis or LIP have a restrictive or mixed pattern spirometry. Interstitial pneumonitis, LIP and PCP are likely to lead to a reduced diffusion capacity for carbon monoxide (DLCO).

Comprehensive lung function measures are therefore needed to delineate the spectrum of CLD, monitor progression, and guide therapy and treatment response. These include measurements of lung capacities and flow, such as spirometry and bronchodilator response testing; measurement of lung volumes with plethysmography; measurement of resistance and compliance with tests such as the forced oscillation technique (FOT), interrupter technique or single-breath occlusion technique; measurement of gas diffusion with single-breath carbon monoxide lung diffusion test to assess alveolar-capillary membrane function; measurement of ventilation distribution with multiple breath nitrogen wash-out test (MBW); and cardiopulmonary functional assessment with the six-minute walk test (6MWT) and exercise (treadmill) testing.

The aim of this study was to review the available data on the spectrum and determinants of lung function abnormalities in perinatally HIV-infected children and adolescents.

## Methods

A review of published literature was performed by searching “PUBMED”, “African wide” and “CINAHL” via EBSCO Host using the MeSH terms “Respiratory function” AND “HIV” OR “Acquired Immunodeficiency Syndrome” AND “Children” OR “Adolescents”; full search terms are shown in Table 1. The search was limited to English language articles with a publication date between January 1984 and September 2017. Articles involving infants, children, adolescents or youth, HIV-infected or exposed, and lung function testing were included. Articles on adult studies or healthy populations were excluded. Where full articles could not be retrieved on Endnote, the full article was requested from the corresponding author by email. In addition to database searches, other relevant references from previous original articles were searched manually through Google Scholar. Data regarding patient characteristics, lung function test used and outcome were abstracted and summarized in table format.

**Table 1:** Search strategy for review of lung function in HIV-infected children and adolescents

Database	MeSH	Key words
PUBMED	Respiratory Function Tests	lung function test OR pulmonary function test OR Respiratory Function Tests
	HIV OR Acquired Immunodeficiency Syndrome	HIV OR human immunodeficiency virus OR AIDS OR Acquired immunodeficiency syndrome OR Acquired Immuno-deficiency Syndrome or Acquired Immunodeficiency Syndrome
		Children OR pediatric OR paediatric OR neonates OR Adolescents OR teenagers OR youth OR young people OR infants
		Search, Query, Items found, Time
		#35, “Search (((((““Respiratory Function Tests””[Mesh]) OR (((Respiratory Function Tests) OR pulmonary function test) OR lung function test))) AND ((((““Acquired Immunodeficiency Syndrome””[Mesh]) OR ““HIV”“[Mesh])) OR ((((((HIV) OR human immunodeficiency virus) OR AIDS) OR Acquired immunodeficiency syndrome) OR Acquired Immuno-deficiency Syndrome) OR Acquired Immunodeficiency Syndrome)))) AND (((((((((Children) OR pediatric) OR paediatric) OR Adolescents) OR youth) OR young people) OR infants) OR teenagers) OR neonates) Sort by: [relevance]”, 146,08:58:16
		#34, “Search (((““Respiratory Function Tests””[Mesh]) OR (((Respiratory Function Tests) OR pulmonary function test) OR lung function test))) AND ((((““Acquired Immunodeficiency Syndrome””[Mesh]) OR ““HIV”“[Mesh])) OR ((((((HIV) OR human immunodeficiency virus) OR AIDS) OR Acquired immunodeficiency syndrome) OR Acquired Immuno-deficiency Syndrome) OR Acquired Immunodeficiency Syndrome)) Sort by: [relevance]”, 659,08:54:18
		#33, “Search ((((((((Children) OR pediatric) OR paediatric) OR Adolescents) OR youth) OR young people) OR infants) OR teenagers) OR neonates Sort by: [relevance]”, 4,074,285,08:53:22
		#32, “Search infants Sort by: [relevance]”,1,118,057,08:51:23
		#31, “Search young people Sort by: [relevance]”, 805,914,08:51:04
		#30, “Search youth Sort by: [relevance]”, 1,839,127, 08:50:35
		#29, “Search teenagers Sort by: [relevance]”, 1,822,104, 08:50:16
		#28, “Search Adolescents Sort by: [relevance]”, 1,843,588, 08:49:54
		#27, “Search neonates Sort by: [relevance]”, 572,698, 08:49:40
		#26, “Search paediatric Sort by: [relevance]”, 413,775, 08:49:16
		#25, “Search pediatric Sort by: [relevance]”, 612,753, 08:48:58
		#24, “Search Children Sort by: [relevance]”, 2,174,198,08:48:33
		#23, “Search (((““Acquired Immunodeficiency Syndrome””[Mesh]) OR ““HIV”“[Mesh])) OR ((((((HIV) OR human immunodeficiency virus) OR AIDS) OR Acquired immunodeficiency syndrome) OR Acquired Immuno-deficiency Syndrome) OR Acquired Immunodeficiency Syndrome) Sort by: [relevance]”, 424,852, 08:47:05
		#22, “Search (((((HIV) OR human immunodeficiency virus) OR AIDS) OR Acquired immunodeficiency syndrome) OR Acquired Immuno-deficiency Syndrome) OR Acquired Immunodeficiency Syndrome Sort by: [relevance]”, 424,852, 08:46:36
		#21, “Search Acquired Immunodeficiency Syndrome Sort by: [relevance]”, 88,073, 08:44:20
		#20, “Search Acquired Immuno-deficiency Syndrome Sort by: [pubsolr12]”, 88,141, 08:44:00
		#19, “Search Acquired immunodeficiency syndrome Sort by: [relevance]”, 88,073, 08:43:37
		#18, “Search AIDS Sort by: [relevance]”, 250,617, 08:43:14
		#17, “Search human immunodeficiency virus Sort by: [relevance]”, 328,144, 08:42:43
		#16, “Search HIV Sort by: [relevance]”, 316,415, 08:42:22
		#15, “Search (““Acquired Immunodeficiency Syndrome””[Mesh]) OR ““HIV”“[Mesh] Sort by: [relevance]”, 152,381, 08:41:48
		#14, “Search (““Respiratory Function Tests”“[Mesh]) OR (((Respiratory Function Tests) OR pulmonary function test) OR lung function test) Sort by: [relevance]”, 224,085,08:40:33
		#13, “Search ““Respiratory Function Tests”“[Mesh] Sort by: [relevance]”, 213,706,08:40:04
		#12, “Search ((Respiratory Function Tests) OR pulmonary function test) OR lung function test Sort by: [relevance]”, 224,085,08:38:54
		#11, “Search Respiratory Function Tests Sort by: [relevance]”, 218,117, 08:38:08
		#10, “Search pulmonary function test Sort by: [relevance]”, 222,124, 08:37:37
		#9, “Search lung function test Sort by: [relevance]”, 221,928, 08:37:11
		#8, “Search ““Acquired Immunodeficiency Syndrome”“[Mesh] Sort by: [relevance]”, 74,419,08:36:14
		#4, “Search ““HIV”“[Mesh] Sort by: [relevance]”, 88,572,08:29:34
African wide and CINAHL via EBSCO Host		lung function OR lung function test* OR pulmonary function OR pulmonary function test* OR Respiratory Function OR Respiratory Function Test*
		HIV OR human immunodeficiency virus OR AIDS OR Acquired immunodeficiency syndrome OR Acquired Immuno-deficiency Syndrome or Acquired Immunodeficiency Syndrome
		Child* OR pediatric OR paediatric OR neonat* OR Adolescen* OR teenage* OR youth OR young people OR infant*OR young adult*

## Results

**Fig. 1:**
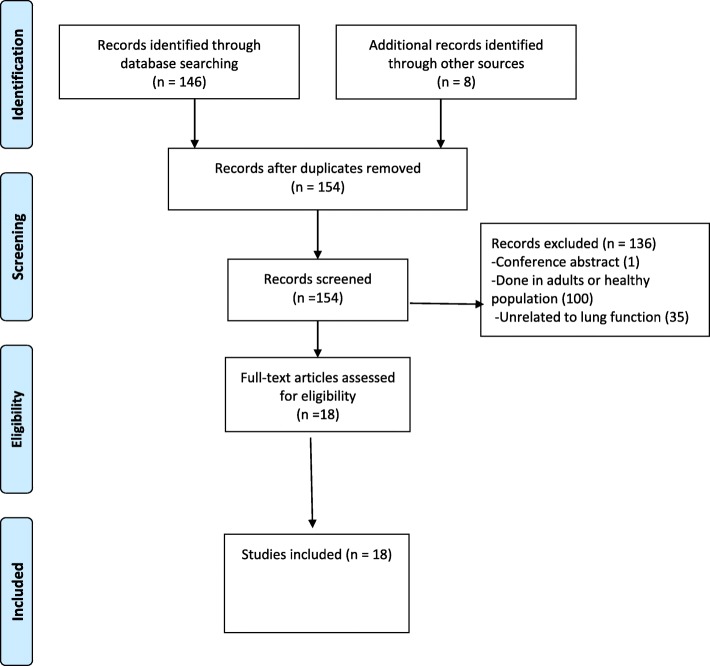
PRISMA Flow Diagram

The process of the literature search is shown in Fig. 1. After combining all the search terms, 146 articles were found; 8 additional articles were obtained by a manual search, (Fig. 1). One hundred and thirty-six studies were excluded because they were unrelated to lung function, or were not related to the population of interest, or only a conference abstract was available. Eighteen full-text articles were found and included in this review (Table 2). All included studies were published between July 1997 and September 2017. Of the 18 included studies, 11 were from Africa, 6 from the United States of America (USA) and 1 from Italy. Three studies focused on infants (two of which also included HIV-exposed uninfected infants) [7–9], two focused on children < 8 years [10, 11] and 13 focused on adolescents and youth (9–24 years), (Table 2). Eleven studies had a comparator group (control) (Table 2). All the HIV-infected participants were perinatally infected.

**Table 2:** Summary of studies on lung function in HIV-infected children and adolescents

Author, Journal	Symptoms	Study design &country	Participant characteristics	Lung function test	Summary of results
Desai et al. [5] 2017 Clin Infec Dis	−25% chronic cough − 5% wheeze −18% resting hypoxia	Cross-sectional, Zimbabwe	HIV-infected adolescents, median age 11 years, *n* = 193, ART duration 5 years	Spirometry with BDR	-Mosaic attenuation and bronchiectasis on HRCT strongly correlated with FEV_1_, *r* = −0.52, and *r* = −0.50, *p* < 0.001 respectively.
Shearer et al. [20] 2017 J Allergy Clin & Immuno	−34% had history of physician-diagnosed asthma	Cohort, USA	218 HIV-infected, all on ART; 152 HIV-uninfected exposed; median age 17 years	Spirometry with BDR	-Obstructive spirometry pattern similar in both groups (22% vs 21%). −17% HIV-exposed uninfected youth had positive BDR vs 9% in HIV-infected youth, *p* = 0.052
Githinji et al. [12] 2017 Annals of ATS	−10% had history of asthma −4% had clubbing − 15% anytime cough	Cohort study, South Africa	515 HIV –infected adolescents, median age 12 years; mean ART duration 8 years, and 110 HIV-uninfected	Spirometry with BDR, FOT, N_2_MBW, Single breath CO 6MWT	-Flow, volume, compliance, diffusion capacity lower in HIV-infected than uninfected; Higher resistance and LCI in HIV-infected compared to uninfected, *p* < 0.05 -No cardiorespiratory function impairment on exercise testing in both groups
Gray D. et al. [7] 2017 Thorax	–	Birth cohort	129 infants HIV-exposed uninfected; 546 infants born to HIV-uninfected mothers; median age 50 days	Tidal breathing and flow volume loops	-HIV-exposed infants had higher tidal volumes compared to infants born to HIV-uninfected mothers, *p* = 0.04
McHugh et al. [1] 2016 AIDS	−54% chronic cough −16% reported dypnoea	Cross-sectional, Zimbabwe	385 HIV-infected children, median age 11 year, none on ART	Spirometry with BDR, shuttle walk test	−10% obstructive spirometry; 1.3% BDR −18% reduced FVC − 10% desaturated to < 88% on exercise
Rylance et al. [15] 2016 Arch dis child (poster abstract)	-Those receiving ART, 15% had dyspnea −15% had daily cough	Cross-sectional	385 HIV-infected ART-naïve;202 on ART; median age 11 years	Spirometry 6MWT	-Proportion of abnormal spirometry similar in ART-exposed and ART-naïve group (25.6% vs 24.3%) -Less distance in 6MWT in ART-naïve group, *p* < 0.001
Mwalukomo et al. [13] 2016 Peds Inf Dis	−8% had history of wheeze − 22% had finger clubbing − 20% had resting hypoxia	Cross-sectional, Malawi	160 HIV-infected; median age 11 years 71% on ART median duration 3.5 years	Spirometry with BDR	-18% obstructive spirometry, 20% reduced FVC; 32% had + BDR
Rylance et al. [16] 2016 AIDS	− 15% had chronic cough − 15% had dyspnea − 5% had wheeze	Cross-sectional, Zimbabwe	150 HIV-uninfected;202 HIV-infected;median age 11 years ART mean duration 5 years	Spirometry with BDR, Shuttle walk test	-Lower FEV_1_, FVC, and FEF_50_ in HIV-infected, *p* < 0.05. 11 (35%) out of 31 with obstructive spirometry had + BDR -Less distance walked in HIV-infected, *p* < 0.001
Chisati et al. [17] 2015 Malawi Med. Journal	–	Cross-sectional, Malawi	55 HIV-infected youth, not on ART and 78 uninfected youth, mean age 24 years	Treadmill exercise test	-Lower VO_2_max (aerobic endurance) in HIV-infected compared to uninfected, *p* = 0.01
Masekela et al. [14] 2012 Int J Tuberc Lung Dis	–	Cross-sectional, South Africa	35, 6-18y with HIV-related bronchiectasis, all on ART	Spirometry with BDR	-Median FEV_1_ was 53%
Ferrand et al. [6] 2012 Clin Inf Dis	−35% resting hypoxia −66% recurrent cough − 10% clubbing	Cross sectional, Zimbabwe	116 adolescents mean age 14 years, vertically HIV-infected, 69% ART mean duration 20 months	Spirometry with BDR, 200 m brisk walk	-45% had FEV_1_ < 80%; 47% had CXR abnormalities, 55% had mosaic attenuation on HRCT
Samadi et al. 2012 (unpublished data)	–	Cross-sectional, South Africa	56 HIV infected on INH prophylaxis, 7-10y, none on ART	Spirometry with BDR	−21% had abnormal spirometry; 18% had positive BDR
Cade et al. [29] 2002 Ped Rehab		Cross-sectional, USA	15 HIV-infected adolescents,14 on ART &15 matched HIV-uninfected, median age 18 years	Treadmill exercise test	-Peak oxygen consumption, treadmill duration and oxygen pulse were lower in HIV infected adolescents compared to uninfected, *p* < 0.05 for all
Colin A et al. [9] 2001 AJRCCM	–	Cohort, USA	285 HIV-exposed uninfected infants born to HIV-infected mothers, 92 HIV-unexposed uninfected infants	Vmax FRC by rapid thoracic compression	-Forced expiratory flow was ≈20% less in the HIV-exposed group but this difference was non-significant
Keyser et al. [30] 2000 Arch Phys Med Rehabil	–	Cross-sectional, USA	17 HIV-infected mean age 18 years; all on ART	treadmill exercise test	-Peak oxygen consumption was lower than expected (functional 2aerobic impairment)
Platzker et al. [8] 2000 AJRCCM	–	Cohort, USA	41 infants born to HIV-infected mothers (34% of infants HIV-infected), mean age 24 months	Thoraco-abdominal compression	-Respiratory system compliance reduced and declined more after TAC in HIV-infected, *p* = 0.003 -Higher resistance in HIV-infected infants compared to uninfected, *p* = 0.03
Alderson et al. [10] 1999 Radiology	–	Cohort, USA	132 HIV-infected children, mean age 47 months and 160 HIV-exposed uninfected infants; mean age 10 months	Lung diffusion capacity using ^99m^Tc DTPA	-HIV-infected children had faster clearance of ^99m^Tc DTPA compared to HIV-exposed uninfected children, *p* < 0.05, in the absence of clinical symptoms
De Martino et al. [11] 1997 Paeds Pulm	–	Prospective longitudinal cohort, Italy	54 children, median age 64 months, with perinatal HIV infection, none on ART and 315 healthy controls	Interrupter technique	-Airway resistance greater in HIV-infected than uninfected, *p* < 0.001

*+BDR* Positive bronchodilator responsiveness in FEV_1_ > 12%, HEU-HIV-exposed uninfected, *FEV*_*1*_ Forced expiratory volume in 1 s, *FVC* Forced vital capacity, *FEF*_*2575*_ Forced expiratory flow between 25 and 75 s of vital capacity, ^*99m*^*Tc DTPA* Diethylene triamine pentaacetic acid, *6MWT* Six-minute walk test, *FOT* Forced oscillation technique, *N*_*2*_*MBW* Nitrogen multiple breath wash-out test, *CO* Carbon monoxide, *HRCT* High resolution chest tomography

Baseline characteristics of participants differed among studies with median age ranging from 50 days to 24 years. The number of participants in each study ranged from 100 to 600, with a total of 2051 HIV-infected participants pooled from all studies. Severity of disease differed; Ferrand et al. [6] reported 66% had chronic cough, McHugh et al. [1] reported 54% had chronic cough. Githinji et al. [12] reported 3.5% had clubbing while Mwalukomo [13] reported 22% with digital clubbing.

Participants were reported to have been on ART in 75% of the studies (Table 2). The duration of ART was reported in 5 studies and ranged from 2 to 8 years [12, 14–16]. In 3 studies, no participant was on ART (Table 2); 2 of these studies took place in sub-Saharan Africa [1, 17] and 1 in Italy in the pre-ART era [11].

Lung function measures reported were spirometry with bronchodilator response testing and exercise testing (with treadmill or incremental shuttle walk test or 6MWT). One study included comprehensive lung function testing including FOT and MBW tests [12].

Spirometry testing was standardized in all studies as per American Thoracic Society (ATS)/European Respiratory Society (ERS) criteria [18]. The definition of restrictive pattern spirometry varied across studies with most reporting reduced forced vital capacity (FVC) as a spirometry pattern. The definition of obstructive pattern also varied across studies, with some studies using the lower limit of normal of forced expiratory volume in 1 s/forced vital capacity (FEV_1_/FVC), as per the global lung initiative reference [19] and others using FEV_1_/FVC < 80%. Shearer et al. [20] had broad inclusion criteria of obstructive spirometry pattern including FEF_25–75_ < 65% or FEV_1_/FVC < 80%.

Assessment and definition of bronchodilator responsiveness varied among the studies. Criteria for bronchodilator responsiveness (BDR) in most studies was change in FEV_1_ > 12%. Shearer et al. [20] used albuterol and a change of ≥10% in FEV_1_. Three studies used 2.5 mg nebulized salbutamol [1, 13, 16] while the rest used 400 μg inhaled salbutamol.

Of the 10 studies reporting spirometry findings (Table 2), 9 reported obstructive spirometry pattern, 6 of which demonstrated low rates of bronchodilator reversibility. In 5 studies with a comparator group, this rate of irreversible obstruction spirometry was higher in the HIV-infected. Rylance et al. [16] reported 11 (35%) out of 31 HIV-infected children with obstructive spirometry had positive bronchodilator responsiveness, while Githinji et al. [12] reported 15% of HIV-infected adolescents had bronchodilator responsiveness compared to 8% HIV-uninfected adolescents (*p* = 0.058). Mwalukomo et al. [13] reported 31.9% of the HIV-infected participants had bronchodilator responsiveness. Shearer et al. [20] reported similar rates of obstructive pattern spirometry between HIV-infected youth and HIV-exposed uninfected youth (22% vs 21%), but a lower rate of bronchodilator responsiveness in the HIV-infected youth (17% vs 9%, *p* = 0.05).

Two studies reported diffusion tests (Table 2) that were found to be lower or impaired in the HIV-infected group compared to the uninfected. Airway obstruction and reduced diffusion capacity were consistent findings across age-groups from childhood [10, 11] to adolescence (Table 2).

Seven studies reported exercise tests for cardiopulmonary function status (6MWT or treadmill test) (Table 2), which showed that HIV-infected participants had functional aerobic impairment except for 1 study where no difference in distance walked or oxygen desaturation was reported after exertion (Table 2).

Determinants of lung function were reported in 4 studies [5, 6, 12, 13]. History of previous lower respiratory tract infection or pulmonary TB was associated with reduced FEV_1_ and DLCO [12]. Cough > 1 month was 2.9 times more likely to be associated with abnormal spirometry (95%CI 1.21–7.10) [13]. Mosaic attenuation and extent of bronchiectasis were significantly associated with reduced FEV_1_, (*r* = − 0.52 and *r* = − 0.50, *p* < 001, respectively) [5].

One study reported MBW and FOT besides spirometry (Table 2), where HIV-infected adolescents had increased resistance, lower compliance, reduced functional residual capacity and increased lung clearance index compared to HIV-uninfected adolescents.

Two studies involved HIV-exposed uninfected children [7, 9], and 1 study had HIV-exposed uninfected youth as a comparator group [20]. Forced expiratory flow was about 20% less in the HIV-exposed group but this difference was not significant [21].

A summary of all studies included in this review is presented in Table 2. Overall, results showed that HIV-infected participants had reduced flow and volume and functional aerobic impairment on exercise, reduced compliance, increased respiratory system resistance and reduced diffusion capacity compared to HIV-uninfected participants. Participants who had longer ART duration had less severe respiratory symptoms, less severe lower airway obstruction and no aerobic impairment.

## Discussion

This review provides evidence of impairments in lung function in perinatally HIV-infected children and youth—predominantly irreversible lower airway obstruction, reduction in exercise tolerance and reduced diffusion capacity [1, 6, 15, 16]. Fixed airflow obstruction was the most commonly reported finding, irrespective of ART status (Table 2).

Irreversible airway obstruction is likely to be a response to airway epithelium injury by opportunistic infections (OIs) or from HIV, repair of which can lead to proliferation of granulation tissue, fibrosis of airways and subsequent obliteration of the lumen [22]. Bronchiolitis obliterans, which may result in irreversible lower airway obstruction, has been reported as a predominant pathology, evidenced by radiological manifestation of mosaic attenuation on chest tomography in HIV-infected adolescents with delayed access to ART [5, 6]. Systemic inflammatory markers have also been found to be increased in uncontrolled HIV or following repeated infections [23]. Lung infections like PCP [24] have been associated with increased metalloproteinases and chronic airflow obstruction in adults but none of the studies in this review reported prior PCP in participants.

ART has been reported in HIV-infected adults to be independently associated with irreversible airway obstruction but the mechanism remains unproven [25, 26]. A direct effect of ART on inflammation in the lung and airways by reduction of peroxisome proliferator-activated receptor has been reported in adults [27]. Bronchodilator reversibility was reported to be present in 15–35% of participants. Despite the available evidence that irreversible airway obstruction is common in HIV-infected children, use of inhaled asthma medications has been reported to be widely used in HIV-infected children and adolescents [28]. Although bronchodilator reversibility was more common in HIV-exposed uninfected youths than in the HIV-infected youths, Shearer et al. [20] reported that self-reported asthma diagnosis was higher in HIV-infected youths than uninfected. This may be due to constellation of symptoms of asthma-like respiratory illness; for example, wheeze and cough in HIV-infected population and physician use of inhalers in the patients who present with such symptoms.

Differences in disease severity across study populations were more likely a result of varying duration of HIV infection and ART use. Those who had ART therapy for a duration of more than 7 years reported lower prevalence of respiratory symptoms [12] than those who initiated ART in later childhood [1, 6]. Chronic lung diseases like bronchiectasis and bronchiolitis obliterans are likely to have occurred by the time of ART initiation, with most of these studies reporting higher prevalence of chronic cough and wheeze [1, 5, 6].

Functional aerobic impairment was more common in HIV-infected participants than uninfected [1, 16, 17, 29, 30]. Those on ART were reported to have done better on exertion than those not on ART [15]. The exercise intolerance may have been due to impaired ventilation-perfusion mechanics with possible heart dysfunction, though no study in this review reported cardiac function. The cardio-pulmonary function status results across studies were inconsistent due to patient selection differences, with Githinji et al. [12] reporting no significant difference in exercise status between the HIV-infected adolescents and the uninfected, and Chisati et al. [17] reporting low aerobic endurance in the HIV-infected group. These differences may be explained by differences in the cohorts and ART use; all children in the former cohort were stable on ART for a median duration of 8 years, whereas none of the youth in the latter study were on ART.

While adult studies [25, 31] have reported diffusion impairment as predominant lung function abnormality, diffusion impairment in HIV-infected children and adolescents has not been commonly investigated. However, HIV-infected adolescents on ART were reported to have lower diffusion capacity compared to HIV-uninfected adolescents [12]. This suggests that HIV or opportunistic infections may impair oxygen diffusion either by thickening of alveolar-capillary membrane due to interstitial inflammation or post-inflammation fibrosis, or due to reduced surface area for gas exchange, as seen in HIV-related bronchiectasis or bronchiolitis obliterans. Alveolar-capillary membrane integrity may be damaged by HIV and/or opportunistic infections well before the presence of clinical symptoms, as reported by Alderson et al. [10]. Emphysema, unlike in adults, was not documented as a common presentation in HIV-infected adolescents in Zimbabwe [5, 6]. Inflammation of the alveolar-capillary membrane by opportunistic infections like PCP and other acquired immunodeficiency syndrome (AIDS)-related complications has also been documented [31, 32]. Low diffusion capacity has been reported in adult patients who had previous TB in a South African cohort [33]. One study reported that pulmonary TB was associated with reduced DLCO in HIV-infected adolescents [12].

The evidence on the impact of HIV in utero is evolving, with only a few studies to date investigating HIV-exposed uninfected infants. These found no difference in spirometry pattern or forced expiratory flow on thoraco-abdominal compression between HIV-unexposed infants and HIV-exposed uninfected infants [9, 20]. Gray [7], however, reported increased tidal volumes in HIV-exposed uninfected infants compared to unexposed infants soon after birth.

Although accelerated lung function decline has been shown in HIV-infected adults [34, 35], published data on longitudinal lung function changes in HIV-infected children and adolescents are lacking. HIV has been reported to cause decline in lung function after controlling for other respiratory infections [31]. Bacterial pneumonia in HIV has been associated with permanent declines in FEV_1_, FVC, FEV_1_/FVC and DLCO [36]. Pneumonia and pulmonary TB were reported to be more common in HIV-infected adolescents than uninfected in two of the studies [12, 16]. A result of prenatal and postnatal origin of adult chronic obstructive airway disease as reported in several studies [7, 37, 38] remains to be proven by longitudinal studies where these HIV-infected children and adolescents are followed to adulthood.

Limitations of this review include heterogeneity among studies and lack of reporting by some studies on the duration of ART. The studies were also carried out in different eras of PMTCT and ART roll-out, where ART was initiated at varying CD4 counts or clinical stages [39]. Description of obstructive and restrictive spirometry patterns was not uniform across studies with most studies reporting reduced FVC as a spirometry pattern and 1 study also including FEF_25–75_ in the definition of obstructive spirometry_._ Determinants of lung function were also not widely reported. Almost all studies were cross sectional with very limited data on longitudinal changes in lung function over time.

## Conclusion

There is increasing evidence that HIV-infected children and adolescents have high prevalence of lung function impairment, predominantly irreversible lower airway obstruction and reduced aerobic function. Lung function impairment was milder in cohorts of adolescents/children who had had earlier access to ART. Lung function impairment starts early in life in the absence of ART, as evidenced by the papers published in the pre-ART era. Achievement of viral suppression through ART may preserve lung function, though at a lower level compared to HIV-uninfected individuals [1, 12, 20, 34].

## Future directions

There is a need for longitudinal studies on lung function in HIV-infected children and adolescents in the post-ART era into adulthood, as there is increasing evidence that chronic obstructive pulmonary disease has its origins in early life [38]. There is also need for more studies comparing lung function among HIV-infected, HIV-exposed uninfected and HIV-uninfected children and adolescents, to provide evidence on how exposure to maternal virus in utero may affect lung function and how early intervention with ART in HIV-infected pregnant mothers may help to preserve lung function in infants and children.

## Abbreviations

6MWT: Six-minute-walk test
ART: Antiretroviral therapy
BDR: Bronchodilator responsiveness
CLD: Chronic lung disease
DLCO: Single breath diffusion test for carbon monoxide
FOT: Forced oscillation technique
MBW: Nitrogen multiple-breath washout test
PCP: *Pneumocystis jirovecii* pneumonia
PMTCT: Prevention of mother-to-child transmission
